# Caregiver Burden in Informal Caregivers of Patients in Saudi Arabia Receiving Hemodialysis: A Mixed-Methods Study

**DOI:** 10.3390/healthcare11030366

**Published:** 2023-01-28

**Authors:** Bushra Alshammari, Helen Noble, Helen McAneney, Farhan Alshammari, Peter O’Halloran

**Affiliations:** 1 Medical and Surgical Nursing Department, College of Nursing, University of Hail, Hail 2440, Saudi Arabia; 2 School of Nursing and Midwifery, Medical Biology Center, Queen’s University Belfast, 97 Lisburn Rd, Belfast BT9 7BL, UK; 3 UCD Center for Interdisciplinary Research, Education and Innovation in Health Systems, University College Dublin, Belfield, 4 Dublin, Ireland; 4 Department of Pharmaceutics, College of Pharmacy, University of Hail, Hail 2440, Saudi Arabia

**Keywords:** caregivers, carers, burden, hemodialysis, renal failure, kidney diseases, family caregivers, informal caregivers

## Abstract

(1) Background: Long-term caregiving for patients receiving hemodialysis (HD), is associated with physical and psychological stress, which may impact on the well-being and quality of life of caregivers. Due to a lack of understanding of the experiences of informal caregivers of patients receiving HD, especially in Saudi Arabia, this study aimed to measure burden in informal caregivers of patients receiving HD, examine the factors that predict caregiver burden (CB), and explore the experience of burden in caregivers of patients receiving HD. (2) Methods: This study used a mixed-methods, sequential, explanatory design, which consisted of two phases. Phase 1 involved a cross-sectional study design, with a convenience sample of 61 caregivers of patients on maintenance HD for at least 3 months. All caregivers in the study completed the Arabic version of the Zarit Burden Interview to identify caregiver burden. Phase 2 of the study involved a qualitative descriptive design involving semi-structured interviews with nine caregivers. (3) Results: Study findings indicate that caregivers did not experience severe burden. Being older, a female caregiver and having comorbidities was positively associated with increased levels of caregiver burden. In the qualitative phase of the study, a number of important factors emerged that may contribute to a reduction in caregiver burden, including social support, cultural acceptance, and religious influences. (4) Conclusion and impact: CB was found to be low when a comparison was made with other studies using similar populations. Understanding the factors that influence caregiver burden will contribute to the accurate assessment of caregiver burden and help reduce burden in informal caregivers, patients with renal failure, and others with chronic illnesses worldwide.

## 1. Introduction

Informal caregivers play a significant role in providing care to patients with chronic illnesses [1]. Patients with CKD often rely on others to help with medical needs and daily living activities, especially those who require dialysis treatment [2]. Hemodialysis (HD) is considered one of the most distressing treatments impacting patients with CKD in all aspects of life [3,4,5]. Currently, Saudi Arabia and Belgium have the highest prevalence of CKD (24%), followed by the UK and Singapore (16%) [6]. In Saudi Arabia, the number of patients receiving HD increased by 100% during 2007–2017 [7], producing new challenges for health professionals and increasing the burden not only on health-care systems but also on caregivers. However, there is limited research on the burden of care for informal caregivers of patients with CKD in Saudi Arabia and other Middle Eastern countries [8]. While Saudis share a great deal of common cultural aspects with Arabs and Muslims worldwide, Saudi culture is still unique in many ways; for example, men can marry up to four wives at the same time [9]. Saudi also has a conservative Muslim culture with traditional gender roles and an extended family system in which family social support is the norm [9]. The strong family bond inherent in Saudi Arabian culture where siblings are expected to be actively involved in the care, and the collectivism in Saudi society and in Muslim culture in general can influence the experience of caregiving. Culture can have many effects on caregiving expectations and behaviors, such as the definitions of what constitutes “good” and “bad” care, motivations to provide care (why/how people provide care), concepts of caregiver distress or burden, and caregiver illness beliefs (how a caregiver views the care recipient’s health or illness) [10]. Studies need to view CB as a complex phenomenon that is affected by many factors. Increasing awareness and understanding of the cultural context for caregiving is likely to contribute towards achieving the ultimate goal of providing high-quality care to these patients and families in the future.

The Caregiver Stress Theory (CST) [11] provides the theoretical framework for understanding the experiences of caregivers of a chronically ill relative, and the factors that have the greatest influence in predicting CB. The CST proposes that physiological and psychosocial responses to CB are a product of the objective burden of the tasks associated with caregiving, such as hours of care and care arrangements; other existing burdens and stressful life events, as well as social roles and social support; and the caregiver’s cognitive appraisal of the degree of CB [11]. Study findings are discussed within the framework of this theory.

The term “informal caregiver” relates to an unpaid family member, friend, or neighbor who provides care to an individual who experiences illnesses and needs assistance to manage a variety of responsibilities [12]. Caregivers of patients receiving HD may be responsible for supervision of the patient’s nutrition and hygiene, driving patients to treatment sessions, and administering medications [13], and this may have a negative impact on these caregivers [3,4,5], leading to poor health outcomes, feelings of being overwhelmed, social isolation [13], depression, and anxiety [14,15]. These outcomes are moderated by factors including demographic characteristics of both caregivers and care recipients, comorbidity status, cohabiting with the patient, relationship with the patient, duration of caring, and cultural differences [15].

It has been suggested that quantitative measures of CB fail to identify important contextual elements of the caregiving experience and this limitation supports the use of a mixed-methods approach to ensure a more comprehensive understanding of CB. This can strengthen the validity of findings by confirming conclusions from an additional perspective, which reduces the risk of bias, which can occur when using a single research method [16].

Given the high prevalence of CKD in Saudi Arabia and the impact of CB on the well-being of patients and caregivers, there is a need to explore and understand caregiver experiences within Saudi Arabian culture so that appropriate support may be provided.

## 2. Materials and Methods

### 2.1. Aim and Objectives

This study aimed to measure CB in informal caregivers of patients receiving HD, to explore the factors that predict increased CB, and to examine the lived experience of burden in caregivers of patients receiving HD.

### 2.2. Design

The study protocol has been previously published in *BMC Nephrology* [17]. The study used a mixed-methods sequential explanatory approach employing both quantitative and qualitative phases [18]. Quantitative data described the level of CB while qualitative data helped to explain the descriptive findings [19]. Phase 1 sought to answer the first two research questions through a cross-sectional survey method, while phase 2 focused on answering the third research question using a qualitative descriptive design involving semistructured interviews. Data were collected between September 2017 and April 2018 in the dialysis center at King Khaled Hospital, Hail City, in the northern region of Saudi Arabia.

### 2.3. Sample/Participants

Phase 1 of the study used a convenience sample of caregivers of patients receiving HD. Given a medium effect size (f2 = 0.15), significance level of 5, 90% power and 7 predictors, the sample size required for this study was 130 caregivers. The study recruited only caregivers aged over 18 years and patients who had been receiving HD for more than 3 months. Caregivers had to be unpaid, able to communicate, and read and write in English or Arabic. Caregivers were selected by patients, as they could accurately identify the key caregiver providing some level of practical help and support. Caregivers were only recruited into the study when patients consented to their participation. Caregivers who did not meet the eligibility criteria were excluded.

For Phase 2 of the study, we interviewed the caregivers of patients with higher, average, or lower scores on symptom burden measured using the Chronic Kidney Disease Symptom Burden Index (CKD-SBI) [20]. The study employed a qualitative descriptive design involving a number of face-to-face, semistructured interviews with selected caregivers identified by participants during phase 1 of the study. Interviews were continued until data saturation was achieved, and no new themes or subthemes were identified by respondents [21].

### 2.4. Data Collection

#### 2.4.1. Phase 1: Cross-Sectional Study

The Arabic version of the Zarit Burden Interview (ZBI) was used to assess CB [22]. The Arabic ZBI has demonstrated good validity [23,24] and reliability, with Cronbach’s alpha value of 0.97 [25]. The ZBI contains 22 items, which examine five CB domains: burden on the relationship; loss of control over life; finance; social and family life; and emotional well-being. Questionnaire items are rated using a five-point Likert scale, with 0 (rarely) being the lowest score, and 4 (nearly always) being the highest score [26]. The final score is the sum of all response scores for all 22 items. Total scores range from 0 to 88, with 0 to 20 indicating no or slight burden, 21 to 40 suggesting mild burden, 41 to 60 moderate burden, and 61 to 88 severe burden [27]. The ZBI has been used extensively to measure CB in caregivers of patients with a variety of chronic illnesses [23,28] and has recently been used in the study of patients with CKD [2,3,13]. The information sheet and caregiver questionnaire were sent to caregivers in a sealed envelope via the patient. The information sheet included an invitation to contact the researcher should the caregiver have any questions. The sealed envelope also included a separate form asking caregivers whether they were happy to be contacted at a later date to determine if they would like to be interviewed (consent to be contacted form). Questionnaires (if the caregiver wishes, the consent to be contacted form) were returned to the researcher in a sealed envelope by patients or by mail. Returning the questionnaire was judged to imply consent. The questionnaire takes approximately 5 min to complete. The majority of caregivers (67%) completed the questionnaire when they accompanied patients to hospital to receive HD, while the remaining 33% were completed at home by carers and returned to the researcher in a sealed envelope by patients or by mail.

#### 2.4.2. Phase 2: Semi-Structured Interviews

Purposive sampling was used to recruit participants. Patients were identified from groups with the highest, median, and lowest symptom burden scores from the CKD-SBI and were subsequently asked to invite their caregivers to participate in recorded, face-to-face, semi-structured interviews. Participants provided narratives of their experiences providing care to patients receiving HD and any factors influencing experiences of burden. If any caregivers refused to participate in the study, the next caregiver from their group was recruited. Interview questions were formulated based on the CST and following an analysis of the quantitative findings. Interviews lasted approximately 30 to 60 min. All caregivers were interviewed in a private room in the dialysis center that was identified by them as their location of preference.

### 2.5. Ethical Considerations

The study was approved by the Research Ethics Committees, Queen’s University, Belfast, UK, in September 2017, (reference 10.BAlshammari05.17.M6.V1). It was also approved by the Research and Education Centre of King Khaled Hospital, Saudi Arabia, where the study was conducted.

### 2.6. Data Analysis

Quantitative statistical analysis was completed using IBM SPSS Statistics version 22 (SPSS). For scale variables, means and standard deviation were reported for normally distributed data and medians and interquartile range (IQR) for skewed data. For categorical variables, absolute frequency (*n*) and relative frequency (percentage) of responses, such as gender, education level, marital status, and monthly income, were reported. Correlations between pairs of continuous variables were analyzed using Pearson’s correlation coefficient. Multiple linear regression analysis was used for both samples to identify any association between burden scores, demographic characteristics, and other variables. The level of statistical significance was set at *p* < 0.05.

Qualitative data were coded and analyzed using thematic analysis [29]. Audio-recorded interviews were transcribed into Arabic by the researcher and translated by a certified bilingual translator into English. The translator signed a nondisclosure form to guarantee confidentiality of participant data. The data were stored electronically to allow for coding and analysis. Coding was managed using NVivo qualitative data analysis software version 11 [30]. Analysis was undertaken in six phases: familiarization with the data, coding, searching for themes, reviewing themes, defining and naming the themes, and reporting themes [29]. All interviews were coded independently by the research team of PO, HN, and HM, and BA to ensure the trustworthiness of coding. Findings were discussed and verified among the team at each stage to ensure accuracy of interpretation, ensure reliability, and promote rigor. Codes were organized to give greater clarification of the content and allow the generation of overarching themes and meanings [29].

### 2.7. Rigor

The Good Reporting of a Mixed Methods Study (GRAMMS) guidelines was used to assist reporting quality and helped enhance the transparency of study processes [31]. Interviews were translated by a certified bilingual translator and the translation checked for accuracy by the main researcher, BA, a native Arabic speaker.

Data were coded by BA and three of the research team—PO, HN, and HM—independently to ensure dependability and confirmability. Emerging codes and themes were discussed and agreed on by the research team [32]. Member checking was conducted during the interview process. The researcher repeated and rephrased some of the interview questions during the interview in different ways to clarify and ensure understanding of the participants’ answers. All the research team reviewed the transcripts and final report.

## 3. Results

### 3.1. Phase 1: Quantitative Phase

#### 3.1.1. Caregiver Characteristics: Descriptive Statistical Analysis

A total of 61 caregivers completed the questionnaire. Among the respondents, 48 (78.7%) were female, with 55 (90.2%) living in the same residence as the care recipient. The mean age of caregivers was 36 (±12) years (range 19–66 years), which is considered a young population when compared with similar studies completed in Western countries [2,33]. In sum, 23 (37.7%) caregivers were adults and 20 (32.8%) spouses, 34 (55.7%) had attended higher education, 16 (26.2%) had advanced to secondary education, with the remainder receiving only primary school education, 23 (37%) were in full-time employment, 21 (34.4%) were homemakers, 7 (11.5%) were students, and 15 (24.6%) reporting suffering from at least one medical condition (Table 1).

#### 3.1.2. Caregiver Burden

**Figure 1:**
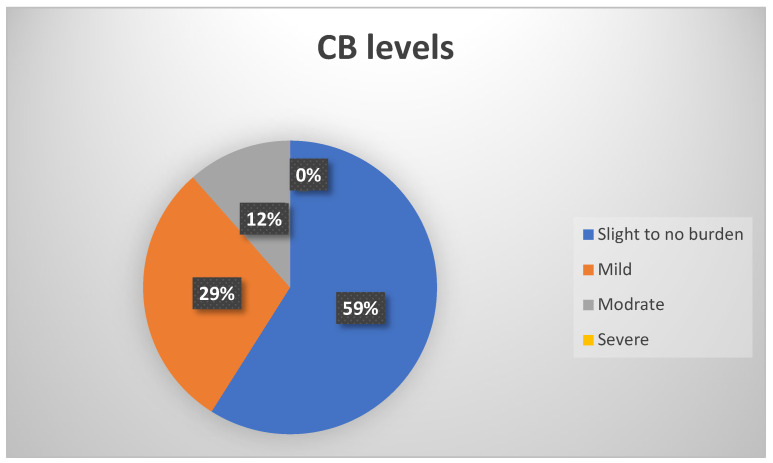
Pie chart presenting the level of burden reported by caregivers.

The mean total burden scores reported by caregivers was 22 (±14), indicating mild to moderate burden (Table 2), while 36 (59%) reported a score of <21, indicating little to no burden, 18 (29.5%) indicated mild burden, and 7 (11.5%) reported moderate burden. No scores provided by study participants indicated feelings of severe burden (Figure 1).

#### 3.1.3. Association between Caregiver Characteristics and Caregiver Burden

CB was found to be positively associated with caregivers’ age, gender, and having a medical condition. Older caregivers reported higher levels of CB (B = 0.553, *p* < 0.001). Being female (B = 0.266, *p* = 0.039) was also associated with increased CB. Caregivers who were spouses or parents of patients receiving HD reported higher burden than sons or daughters who provided care (B = 0.514, *p* < 0.001) (B = 355, *p* = 0.007). Caregivers with at least three medical conditions reported higher CB than those with no conditions. There was a negative association between unemployment and CB (B = −0.240; *p*= 0.074); however, this did not reach a statistically significant level. No significant influence in CB was found in relation to whether caregivers lived with the patient or not. There was no significant correlation between travel duration from home to dialysis centers and burden in caregivers (B = −0.027, *p* = 0.836) (Table 3).

### 3.2. Phase 2: Qualitative Phase

#### 3.2.1. Caregiver Characteristics for Semi-Structured Interview

Caregiver characteristics for those interviewed in the study are presented in Table 4. Fourteen caregivers were invited to take part, of whom one was male. Five declined, and nine female caregivers with different levels of CB were interviewed—four of these were single, four married, and one divorced. The relationship to patients in this population was four daughters, two wives, one sister, one daughter in-law and one mother.

#### 3.2.2. Caregiver Experiences: Thematic Analysis of Qualitative Data

Caregivers were asked a series of open-ended questions focusing on their experience of caregiving, how it impacted their lives, and the factors that contribute to CB in caregivers of patients receiving HD. Following the analysis of this data: three main themes were identified:

1. Positive caregiving experiences
2. Factors influencing CB
3. Negative caregiving experiences

##### Positive Caregiving Experiences

Positive outcomes of providing care to patients were defined as the rewards and satisfaction derived from the caregiving relationship [34]. Caregivers reported that providing care had positive effects, including feeling satisfied and delighted, building a feeling of confidence, and reducing feelings of guilt regarding caring responsibilities for loved ones. Caregivers who were wives expressed feelings of love towards their husbands and dedication to the commitments and responsibilities of marriage. Adult caregivers acknowledged a debt they felt they could never repay to their parents for the love and care they had received from them in early life.

“*Because she is my mother. I owe my parents all my life, and whatever I do for them until my death will not return their favor and efforts*”.

(C7, 35Y, divorced, daughter)

Feeling emotionally fulfilled and satisfied

Caregivers of parents reported being emotionally fulfilled and helpful when they were able to help and care for their parents. A daughter reported that seeing her mother satisfied was a great source of satisfaction for her and provided her with the energy to continue providing care.

“*I have changed for the better. My belief that taking care of my mother is the right thing I should do has been strengthened. Just seeing my mother smiling and satisfied with me makes me feel that I own the whole world*”.

(C7, 35Y, divorced, daughter)

Caregivers reported positive experiences from caregiving, including a sense of giving back to someone who has cared for them, the satisfaction of knowing that their loved one is getting excellent care.

“*I do not consider this a burden whatever happens. She is my children’s grandmother. She loves me and my children. When she was in good health condition, she did us everything and took care of us. Now I take care of her with all love and respect, and I am sure God will reward me for this good deed*”.

(C10, 43Y, married, daughter-in-law)

One wife reported being happy to provide care to her husband, as she did not have the opportunity to spend time with him previously. She reported that after he commenced dialysis treatment, her husband had to retire from work and she was finally able to spend time with him.

“*My husband has retired and I can see him more than before. We became able to spend a lot of time with each other. We live far away from the dialysis center, so I accompany him for a long distance and spend much time with him during treatment sessions. Every cloud has a silver lining. May God help him recover soon*”.

(C6, 40Y, first wife)

Feelings of being useful and appreciated

Some caregivers reported feeling useful and appreciated when they were required to provide care. They indicated that providing care filled free time and gave them a sense of being worthy. Providing care was also a source of motivation to succeed in all aspects of life.

“*Usually, [before her father became ill] I spent my time doing useless things, like sleeping during the afternoon, watching television or playing on my phone, instead of taking care of my father…. In fact, providing care to my father has strengthened my diligence and motivation to be better*”.

(C11, 25Y, single, daughter)

##### Factors Influencing Caregiver Burden

Social support and cultural acceptance

It was notable that when caregivers received social support from family and work organizations, this helped to reduce care burden. One participant reported that she was sharing responsibility for care for her husband with two other wives.

“*I have a lot of time for myself. I sometimes wish to spend more time with him. We (three wives) share in taking care of him*”.

(C6, 40Y, first wife)

Providing care was a collaboration process between sister, brother, and mother in the care of one patient. The brother fulfilled the traveling role, including accompanying his sister (the patient) to the dialysis center and follow-up appointments in and out of the city. The older sister of the patient assumed responsibility for all activities related to physical and emotional care, with the mother proving care during morning when the sister and brother attended university.

“*I take care of her but when she needs to be subjected to some medical checks in another city my brother travels with her as I am tied to my university studies*”.

(C12, 20Y, single, sister)

Informal work support was also provided to the caregivers of patients receiving HD. Daughter caregivers reported that this support involved cooperation between colleagues and managers to afford flexible working arrangements, which helped her to accompany her mother during her afternoon HD sessions. Managers and coworkers at the school where she worked knew of the demands of her caregiving role and were able to assist her by providing flexible teaching schedules and classes with no extra after-school activities, due to increasing care demands. This cooperation helped to reduce work demands and allowed her to provide care more effectively.

“*My employer and colleagues support me. My work schedule is arranged in such a good way where I do not teach the last classes and do not take any school activities at the time when my mother has dialysis sessions, So I am able to leave early and accompany her*”.

(C9, 32Y, single, daughter)

Independence and adaptation of patients

Patients’ symptom burden was reflected in CB throughout the study. Interview data indicated that when patients seemed normal and independent, this reduced the burden on individuals providing care to them. Seeing the patient acting normally provided emotional comfort during distressful periods associated with illness.

“*I do not feel that he is a burden on me nor feel angry with him because love makes one forgive others for a lot of things. My husband makes me feel that life is normal and conveys this impression to me, as a result, this is reflected in my own life. I sometimes forget that we have a patient at home*”.

(C6, 40Y, first wife)

“*I think that taking care of a patient who is active and seemingly healthy with low demand, as in the case of my husband, who gives the impression that he is healthy and not diseased, doesn’t require efforts from me*”.

(C5, 65Y, wife)

Caregiving as a temporary stage in life

Providing care seemed to be a temporary role for most caregivers. One daughter took care of her father, but when she married, her sister continued providing care. Later, the daughter-in-law and grandchildren became more involved. This helped to reduce feelings of being trapped in the caregiving role, which helped reduce emotional strain.

“*My patient’s wives and daughters, were able to know his condition once they see his face. After his daughters married, I began to be involved in taking care of him. I am able to know if he is good or not once I see his face*”.

(C10, 43Y, married, daughter-in-law)

##### Religious Beliefs

It became clear from interview data that the religious and cultural beliefs of caregivers shaped their experiences of caregiving and influenced reported caregiving distress. More religious family members reported being more positive about their role as caregivers and experienced better relationships with those they cared for [35,36]. Caregivers providing care to their parents saw this as a religious commitment.

“*First this is my mother, and I do not do anything worthy of praise. This is my duty and sometimes I feel a bit negligent in this regard. I take care of my mother because our true religion commands us to do so, and because “paradise is under the feet of mothers”. Taking care of my mother brings me luck and success in every step I take. Her satisfaction is an important and essential thing in my life. She needs my help a lot now and I should be beside her*”.

(C9, 32Y, single, daughter)

Religion was an important issue for those providing care to parents. Caregivers undertook significant personal sacrifices in order to meet the expectations of their religious beliefs in achieving the satisfaction of God, who could help them to live in peace and assurance. They believed that God would return their sacrifices with even more joy and blessings in the future.

“*I got divorced from my former husband because I wanted to stay beside (my mother) and take care of her. So it’s unlikely that I will think about marrying again as long as she is in such a difficult condition, all the tiredness I feel vanishes at a smile from my mother or some prayer for me from the depth of her heart wishing me something beautiful to happen. Now I feel inner peace and reassurance I have never felt before*”.

(C7, 35Y, divorced, daughter)

“*I rejected the marriage proposals of a lot of men only to take care of my mother. I asked my brother and sisters who are younger than me to marry and not to wait for me because I don’t want to stand in their way to marry. I have the conviction that my mother is the more important than all, than everything, even myself. She is the cause of my existence and I have to stand by her now. I believe that God hides and saves the most beautiful things for me*”.

(C9, 32Y, single, daughter)

##### Culture

In this study, all caregivers interviewed were women. In Saudi Arabia, the culture typically imposes some social restrictions on women, which limits their activities outside the home. Consequently, some women reported that the caring role did not restrict them socially, as it had little impact on interactions outside the home.

“*My social life has not changed much. We are from a conservative family that does not allow girls to go out with their friends so much*”.

(C12, 20Y, single, sister)

“*Typically, I’m not a social person. I meet my friends at work in the morning and just that*”.

(C9, 32Y, single, daughter)

Negative caregiving experiences

Although some caregivers reported a highly positive caregiving experience, others reported a number of difficulties providing care to patients receiving HD. Caregivers reported that unlike other chronic illnesses, renal failure requires unique treatment as it requires hospital visits at least twice a week for an indefinite period, which can lead to feelings of restriction.

“*The most difficult thing in her illness is that she has a disease that necessitates her to go to the hospital periodically, and that means it cannot be treated at home like other diseases such as diabetes and blood pressure*”.

(C12, 20Y, single, sister)

Travel for recreational or religious purposes became impossible for some because of the need for dialysis.

“*Our lives have become different from the past. We used to travel and go out a lot. I love to travel to Mecca to perform minor pilgrimages from time to time, but after my husband fell ill with this terrible disease and we got tied to treatment plans, I could never go*”.

(C5, 65Y, wife)

Fear was commonly a negative emotional response reported by caregivers. Female spousal caregivers reported being worried about the future for their husbands, and were fearful of taking on new responsibilities.

“*I am afraid that my husband’s condition has worsened. He is my husband and the breadwinner of the family, he holds the family firmly without him I am paralyzed*”.

(C5, 65Y, wife)

Fear of losing patients through death was also reported by a number of respondents. Caregivers were afraid of losing their loved ones, which encouraged them to provide the best care possible to ensure that they did not experience feelings of guilt. Putting themselves under strain by providing excessive care increased feelings of anger and feelings of stress.

“*Sometimes she does not stick to her specific meals or refuses to take some medicines, which makes me feel a bit worried about her and her health condition, in general I rarely get angry and do not even like to consider the idea of being angry in connection with my mother. I do not get angry with my mother, but I am just worried about her and fear living without her*”.

(C7, 35Y, divorced, daughter)

## 4. Integration and Synthesis

In the quantitative phase of the study, CB levels were found to be low compared to similar studies examining individuals receiving HD. Throughout qualitative interviews, caregivers reported a number of positive and therapeutic benefits associated with caregiving. They found satisfaction and meaning when providing care to family members, which was derived from their religious and cultural beliefs. Due to the restricted role of many women, female caregivers found genuine fulfilment in caring, which made them feel productive and worthy. Some caregivers were reluctant to criticize patients, even when they experienced increased burden providing care. They believed that they did not have the right to complain about meeting the care needs of patients, as the patient has a legitimate right to be cared for. Caregivers demonstrated sympathy towards patients and were reluctant to say anything that may be perceived as offensive to patients in their care. Complaining of CB is considered socially and religiously unacceptable, which may result in the underreporting of CB. This was observed in some caregivers who reported the possibility of feeling angry and subsequently denied their own anger. Caregivers prioritized patient well-being and believed that they should focus on the needs of patients, not on their own personal needs. Caregivers believed that if they reported burden to HCPs, this would result in HCPs spending more time meeting needs of caregivers, which would limit their role in providing care to patients.

During the recruitment phase of the study, patients were often unsure who their primary caregiver was, and reported that they received care from a number of people. Patients stated that individuals who drive them to the dialysis center are often different from those who cook for them or help to manage their health needs. Social support played a significant role in reducing physical and psychological burden in caregivers, which indicates that caring responsibilities are shared by a number of family members so that CB does not fall on one individual. Members of the extended family, such as grandchildren, and sons/daughters-in-law, are also involved in providing care. Spreading caregiving tasks to a group of family members reduces the burden on the main primary caregiver. One caregiver interviewed in the study reported that she was one of three wives providing care to a patient, which suggests that unique cultural characteristics and social acceptance can also help reduce the perceived burden experienced by caregivers.

Religion was also reported by respondents as a factor which motivated caregiving, as this was perceived to be a positive function which may enable individuals to be subsequently rewarded by God. Caregivers mentioned various reasons for caring for their patients. Strong faith and “fear of God” were two factors mentioned as empowering for the caregivers, some of whom believed that God would reward them for their efforts. Honoring parents and feeling compassion towards them were the most mentioned religious principles in relation to caring for ill parents.

The age of caregivers and the number of their medical conditions were found to be positively associated with CB in the quantitative analysis. It may be that these caregiver limitations reduce their physical, social, and financial resources, and so their ability to provide care. This in turn may reduce the satisfaction they derive from caring well for their relatives. Female caregivers reported higher CB than male respondents. This may be because caregiving is considered a natural role for women is Saudi Arabia, and therefore they are less likely to receive support from the extended family. Sons and daughters reported less CB than spouses or parent caregivers. Study findings demonstrated that sons and daughters were younger, more energetic, had fewer comorbidities, and were more able physically and mentally to provide care. Sons and daughters believed that providing care to parents was temporary and that in the future other family members will assume this responsibility.

## 5. Discussion

The majority of caregivers reported mild CB, with no scores indicating severe burden. This contrasts with a recent systematic review that reported moderate to severe CB in 24 studies of caregivers providing care to end-stage renal disease patients [15]. Several factors may explain the low CB in the study population, with social support being the most significant contributing factor. The wider literature shows that social support helps to reduce CB in patient receiving dialysis [3,37]. In Saudi Arabia, family support is frequently available, as families are quite large, with a mean family size of 6.4 and a range of 5.5 to 8.4 family members [38]. A large family increases the opportunities for family members to collaborate and share the role of caring so that the CB does not fall entirely on one individual, which helps reduce CB [39]. The strong family bond, collectivism in the Saudi Arabian culture and in Muslims in general [40], the loyalty and willingness to prioritize their family’s needs over one’s own [41], may also contribute to the facilitation of this support. The finding of lower reported CB from the Saudi respondents seems likely to be affected by the interplay between religious pressure, cultural pressure, and traditional female socialization in Saudi culture.

Unlike other studies, the majority of caregivers were sons and daughters, whereas in the literature, spouses were the majority [37]. Young caregivers were involved in caregiving, with a mean age of 36 years, which is considered young in comparison with other studies [37]. Due to the limitation of physical ability to provide care, older age and having comorbid condition is associated with increasing CB, this is consistent with several studies that reported similar findings [42,43,44].

Female caregivers reported positive experiences and being useful and appreciated when providing care to patients. This finding may be explained by many females in Saudi Arabia being unemployed, due to limited acceptable employment options for women within the constraints of Saudi Arabian culture [45]. Finding work without violating the prohibition of unrelated men and women working together in Saudi Arabia is a significant challenge confronting women in the region [45]. Women typically are supported financially by fathers and later after marriage by the husband. The majority of caregivers in this study were women, who were often not in paid employment and therefore had significant free time, especially if unmarried. Providing care is a strategy they use to feel productive and worthy, which may increase feelings of satisfaction. In this context, providing care was more likely to achieve positive emotional outcomes rather than being perceived as a source of stress. This finding is consistent with other studies that reported that older caregivers may feel useful and satisfied due to the sense of meaning that caring creates, the emotional closeness obtained, and added purpose to life [46]. Caregivers demonstrated sympathy for their patients and were reluctant to say anything that may be perceived as offensive to the patients in their care. This finding is supported by caregiver responses during the survey stage of the study. Many caregivers could not understand why the survey used questions that suggested caregiving could be perceived as demanding, such as “Do you feel strained when you are around your relative?” or “How burdened do you feel in caring for your relative”. Some caregivers stated that this survey should not be seen by patients, as this would be upsetting for patients, as they may think they are a burden on their relatives. The ZBI seems to have a number of limitations when used in Saudi Arabian populations and similar limitations may be found in similar cultures within the Middle East. Rather than assessing CB in a universal fashion, the ZBI measure may reflect the culture in which it was developed (the United States). Using a tool like this in an uncritical fashion may risk minimizing the true extent of CB for caregivers in Saudi Arabia or other cultures where social and family responsibilities are of central importance. Interview data showed that there are significant feelings of CB, but this is reluctantly acknowledged by respondents. Tools such as the ZBI may need to be modified to capture the extent and nature of CB in Saudi Arabian and Middle Eastern cultures. Similarly, it is important not to assume that there is only one main caregiver, which may have implications for services to support caregivers.

Religious beliefs provided an important motivation to provide care to sick family members, as this was perceived to be a positive action that would enable individuals to be rewarded by God. This was supported by a similar study in Jordan that reported religious practice and beliefs to be one of the main coping strategies for caregiving [47]. This study also found that cultural differences, traditions, and behaviors can influence the level of reported burden [10], as some patients received care from multiple wives. Muslim culture allows men to be married to up to four women at any one time under specific circumstances [9]. Burden can also be defined differently within diverse cultures. For example, social restrictions may not be a burden to female caregivers in Saudi Arabia, but may be a possible source of burden for caregivers in other regions [48]. Cultural influences were observed in patient behaviors, reflected in them being reluctant to complain about their relative or thinking about leaving the care to someone outside the family. According to values within Middle Eastern countries, care should be provided at home by a family relative and there is a reluctance to move relatives into a nursing home, even if is close to the family residence [49]. Typically, people in Saudi Arabia are reluctant to leave the care of relatives to others or to complain about their caring roles, as to do so would bring social shame or misfortune [49].

The main strength of this study is that it used a mixed-methods approach. This enabled a more holistic investigation of CB as a complex phenomenon, within the unique culture of Saudi Arabia.

The generalisability of findings in this study is limited due to the small sample in both phases of the study and the use of convenience sampling in the quantitative phase. Under half the caregivers invited to participate in the study completed the questionnaire. It was difficult to gain access to caregivers as they often do not present at dialysis centers, and the only method to contact them was through patients receiving HD. The majority of questionnaires were completed in the dialysis center when caregivers were being dropping off, picking up, or waiting for their relatives. It might be that caregivers who did not participate were experiencing higher CB. However, this seems unlikely, as those who did not participate were more likely to be caring for patients who reported lower symptom burden and showed higher level of independence. However, the mixed-methods approach adopted for this study strengths the validity of the findings as it provides a way to validate quantitative findings through the qualitative findings that describe the lived experience of the participants.

Only female caregivers were interviewed in the qualitative phase of the study as no male caregivers participated, which limits our ability to comment on the experiences of male caregivers of patients receiving HD. A further limitation of the study is that in the interview phase of the study, transcripts were translated from Arabic to English. It is possible that the real meanings of Arabic words may have been misinterpreted and accurate meanings lost during translation. Participants often used metaphorical expressions and idiomatic language in Arabic to help highlight experiences of illness and the subsequent impact on their lives. The complexity and the differences between cultures may make understanding these expressions difficult following translation into the target language. The researcher BA attempted to maintain the accuracy of meaning by confirming the accuracy of translated work independently, by asking a bilingual translator to confirm the accuracy of transcription. The researcher provided an explanation of metaphorical and idiomatic language in order to help confirm the emotions and ideas expressed in Arabic, when the principal language of this study was English. This assisted the researcher to engage fully with the participants and provide explanations of their experiences in their primary language.

## 6. Conclusions

Our findings broadly support the concepts of Caregiver Stress Theory, in that CB has been shown to be partly dependent on the objective burden of caring for someone with more severe symptoms, as measured by the CKD-SBI, but is moderated by life events, social roles, social support, and the caregiver’s cognitive appraisal of the degree of CB.

Caregivers reported relatively low levels of CB. This stems partly from a reduction in the burden, as it is shared with others or mitigated through social support, and partly from positive experiences of caregiving, such as enhanced relationships between caregivers and patients, increased family harmony, and a sense of purpose and satisfaction derived from fulfilling obligations to loved ones. This satisfaction was often underpinned by religious beliefs that emphasized God’s approval of caregiving for family members and the reward that caregivers will receive. Whilst these beliefs about the positive value of caregiving may reduce CB, they may also work to reduce reporting of CB, as admitting to negative caregiving experiences may be socially and religiously less acceptable in Saudi Arabian culture. The ZBI (and other measures of socially mediated experiences) may also underestimate the impact of culture on the scores obtained, especially when used in contexts that are very different from those in which they were developed.

Interventions to support informal caregivers of patients receiving HD in Saudi Arabia should take into account the impact of social roles, culture, religion, family structure, and social support, recognizing that caregiving may be shared among family members, is predominantly (although not exclusively) a female role, and that CB may be a positive experience, but may also be underreported. Future research should consider the development of culturally sensitive measurement scales for CB and also explore the experiences of male caregivers.

## Figures and Tables

**Table 1:** Demographic characteristics of caregivers for phase 1.

Variable	Categories	Caregivers (*n* = 61)
Mean age in years (SD)		36.4 (12.9)
Gender, *n* (%)	Male Female	13 (21.3) 48 (78.7)
Educational level, *n* (%)	Illiterate Primary studies Secondary studies Higher education Other	- 11 (18.0) 16 (26.2) 34 (55.7) -
Marital status, *n* (%)	Married Single Widowed Divorced Other	37 (60.7) 18 (29.5) 2 (3.3) 4 (6.6) -
Employment, *n* (%)	Full-time job Part-time job	23 (37.7) -
	Retired	3 (4.9)
	Student (FT & PT)	7 (11.5)
	Unemployed	7 (11.5)
	Housewife	21 (34.4)
	Self-employed	-
Monthly household income (SAR), *n* (%) (1 GBP = 5 SAR)	0–5000 5000–10,000 10,000–15,000 15,000–20,000 20,000–25,000 25,000–30,000 >30,000 missing	9 (14.8) 16 (26.2) 26 (42.6) 8 (13.1) - - - 2 (3.3)
Median dialysis duration in months (IQR)		20 (8–54)
Comorbidities *n* (%)	0 1–2 3–7	46 (75.4) 13 (21.3) 2 (3.3)
Patient Travel time to hospital, minutes. median (IQR)		15 (10–20)
Caregiver relationship to patient *n* (%)	Spouse Sibling Parent Friend Son/daughter Grandchildren Other	20 (32.8) 4 (6.6) 9 (14.8) - 23 (37.7) 2 (3.3) 3 (4.9)
Patients and caregiver live together *n* (%)	Yes No	55 (90.2) 6 (9.8)

Abbreviations: SD, standard deviation; *n*, number; SAR, Saudi Riyal; GBP, British pound sterling.

**Table 2:** Responses of caregivers to ZBI items.

Question	Never *n* (%)	Rarely *n* (%)	Sometimes *n* (%)	Often Frequently *n* (%)	Nearly Always *n* (%)
Do you feel that your relative asks for more help than he or she needs?	26 (42.6)	15 (24.6)	10 (16.4)	7 (11.5)	3 (4.9)
Do you feel that because of the time you spend with your relative you don’t have enough time for yourself?	24 (38.7)	13 (21.0)	14 (22.6)	3 (4.8)	7 (11.3)
Do you feel stressed between caring for your relative and trying to meet other responsibilities for your family or work?	19 (30.6)	10 (16.1)	15 (24.2)	10 (16.1)	7 (11.3)
Do you feel embarrassed about your relative’s behavior?	44 (71)	12 (19.4)	4 (6.5)	1 (1.6)	0 (0)
Do you feel angry when you are around your relative?	48 (77.4)	9 (14.5)	4 (6.5)	0 (0)	0 (0)
Do you feel that your relative currently affects your relationships with others in a negative way?	33 (53.2)	9 (14.5)	14 (22.6)	3 (4.8)	2 (3.2)
Are you afraid of what the future holds for your relative?	5 (8.1)	3 (4.8)	19 (30.6)	10 (16.1)	24 (38.7)
Do you feel your relative is dependent upon you?	47 (75.8)	10 (16.1)	3 (4.8)	1 (1.6)	0 (0)
Do you feel strained when you are around your relative?	41 (66.1)	12 (19.4)	5 (8.1)	2 (3.2)	1 (1.6)
Do you feel your health has suffered because of your involvement with your relative?	41 (67.2)	8 (13.1)	4 (6.6)	7 (11.5)	1 (1.6)
Do you feel that you don’t have as much privacy as you would like because of your relative?	42 (67.7)	12 (19.4)	5 (8.1)	2 (3.2)	0 (0)
Do you feel that your social life has suffered because you are caring for your relative?	26 (41.9)	14 (22.6)	11 (17.7)	2 (3.2)	8 (12.9)
Do you feel uncomfortable about having friends over, because of your relative?	38 (61.3)	15 (24.2)	6 (9.7)	0 (0)	2 (3.2)
Do you feel that your relative seems to expect you to take care of him/her, as if you were the only one he/she could depend on?	26 (42.6)	9 (14.8)	15 (24.6)	5 (8.2)	6 (9.8)
Do you feel that you don’t have enough money to care for your relative in addition to the rest of your expenses?	39 (62.9)	9 (14.5)	6 (9.7)	0 (0)	7 (11.3)
Do you feel that you will be unable to take care of your relative for much longer?	47 (75.8)	9 (14.5)	1 (1.6)	2 (3.2)	2 (3.2)
Do you feel you have lost control of your life since your relative’s illness?	41 (66.1)	8 (12.9)	8 (12.9)	1 (1.6)	3 (4.8)
Do you wish you could just leave the care of your relative to someone else?	45 (72.6)	8 (12.9)	6 (9.7)	2 (3.2)	0 (0)
Do you feel uncertain about what to do about your relative?	24 (38.7)	13 (21)	14 (22.6)	6 (9.7)	4 (6.5)
Do you feel you should be doing more for your relative?	12 (19.4)	9 (14.5)	19 (30.6)	9 (14.5)	12 (19.4)
Do you feel you could do a better job in caring for your relative?	8 (12.9)	11 (17.7)	21 (33.9)	10 (16.1)	11 (17.7)
**Question**	**Not at all**	**A little**	**Moderately**	**Quite a bit**	**Extremely**
Overall, how burdened do you feel in caring for your relative?	20 (32.3)	17 (27.4)	11 (17.7)	9 (14.5)	4 (6.5)

**Table 3:** Association between total caregiver burden and caregiver characteristics.

Predictors	Caregivers Burden		
	Mean SD of Caregiver Burden Level	B	*p* Value
Age		0.553	0.000
Gender Male Female	13 ±7 21 ± 48	**Ref** 0.266	0.039
Education Primary Secondary Higher education	18 ± 15 15 ± 8 22 ± 13	**Ref** −0.105 0.127	0.545 0.465
Marital status Married Single Divorced Widow	21 ± 13 13 ± 10 19± 10 38 ± 4	**Ref** 0.298 −061 0.257	0.019 0.619 0.039
Employment Full time job Retired Student Housewife Unemployed	18 ± 10 24 ± 21 14 ± 10 25 ± 14 9 ± 10	**Ref** 0.072 −0.111 0.225 −0.240	0.571 0.403 0.107 0.074
Household income in SAR 0–5000 5000–10,000 10,000–15,000 15,000–20,000	21 ± 17 16 ± 14 20 ± 12 19 ± 10	**Ref** −0.194 −0.038 −0.074	0.275 0.833 0.644
Comorbidity No comorbidity One or two comorbidities Three or more Cohabiting status Yes No	17 ± 12 24 ± 13 38 ± 4 20 ± 13 15 ± 10	**Ref** 0.227 0.304 **Ref** −097	0.069 0.016 0.458
Travel duration from home to dialysis/min	15 min, range (10–20)	0.027	0.836
Relationship to patient Son and daughter Parent Spouse Sibling Grandchildren Other relationship	12 ± 8 24 ± 13 25 ± 15 22 ± 9 16 ± 9 20 ± 2	**Ref** 0.355 0.514 0.220 0.057 0.158	0.007 0.000 0.078 0.639 0.197

*β* = Beta or standardized regression coefficient, Min = minutes, HG = hemoglobin, SAR = Saudi Riyal, GBP = British pound sterling.

**Table 4:** Demographic information for caregivers who were interviewed.

Carer ID	Pt ID	Age	Sex	Marital Status	Education Level	Occupation	Caregiver Relationship to Patients	Level of CB	Scores of CKD-SBI
C3	P3	21	F	Single	Secondary	Student	Daughter	Mild	Highest
C4	P4	45	F	Married	Primary	Housewife	Mother	Moderate	Highest
C5	P5	65	F	Married	Primary	Housewife	Wife	Moderate	Highest
C6	P6	40	F	Married	Higher education	Housewife	First wife	Mild	Middle
C7	P7	35	F	Divorced	Secondary	Unemployed	Daughter	Mild	Middle
C9	P9	32	F	Single	Higher education	Full time teacher	Daughter	No burden	Middle
C10	P10	43	F	Married	Secondary	Housewife	Daughter in-low	Mild	Lowest
C11	P11	25	F	Single	Higher education	Unemployed	Daughter	Mild	Lowest
C12	P12	20	F	Single	Higher education	Student	Sister	Mild	Lowest

C, caregiver; P, patient; F, female; CB, caregiver burden; CKD-SBI, Chronic Kidney Disease Symptom Burden Index.

## Data Availability

Not applicable.
